# Advantages of Starched Apparatus in Fractures

**Published:** 1854-06

**Authors:** J. S. Gamjee


### *Advantages of the Starched Apparatus in the Treatment of Fractures and diseased Joints.*

BY J. S. GAMJEE, ESQ.

The following notice of Mr. Gamjee's essay, for which the Council of University College have awarded the Liston Clinical Medal, we copy from the *Brit. & For. Med. Chir. Rev.*

This is a practical work, intended to show the beneficial effects of the treatment of fractures by the starched apparatus, as employed of late years by M. Seutin. This apparatus is very simple; splints are made from pasteboard soaked in water, and are then covered, both inside and out, with a thick coating of starch; the limb is bandaged with a roller covered with wet starch, and the splints are then moulded on the limb, all depressions and tuberosities in which are filled or protected with cotton-wool. An outer bandage, also covered with starch, is then applied, and the limb is kept quiet until the whole is dry, which occurs in about 36 hours. When dry, the bandages are slit up in order to see that the application of the splints had been properly performed, and if any swelling of the limb requires a little loosening of the bandage, or if any shrinking of the bandage requires a little tightening. This being done, a bandage starched on the outside is reapplied, and after this has dried, the patient may leave his bed. The great advantages of this plan are, that uniform pressure is applied, reduction is maintained, and confinement to bed for a long period (as common in fractures of the leg, thigh, and femoral neck, treated on other plans) becomes unnecessary. Mr. Gamjee also states that swelling from extravasation of blood, or from inflammation, need not prevent the application of the apparatus if it be judiciously used; and that if the fracture is a compound one, the only difference of treatment is that the wound may be left uncovered by cutting a piece out of the splint. In support of these assertions, 17 cases treated in University College Hospital are related, and some judicious remarks are attached to each. Without going into their analysis, or into the details of the manipulation required for each particular fracture, we may observe that the evidence. as far as it goes, is satisfactory.—*Nashville Journal of Med. & Surgery.*

